# The Classification of Gluteal Augmentation

**DOI:** 10.1055/a-2192-0339

**Published:** 2024-04-04

**Authors:** Ebaa Sabri, Achraf Daoud

**Affiliations:** 1 Department of Plastic Surgery, St. Michael's Clinic, Shrewsbury, United Kingdom; 2 Private Practice, Plastic Surgery, Tunis, Tunisia

Gluteal enhancement is a major growing trend within aesthetic surgery practice. Patient needs include volume augmentation and enhancement of the roundness of buttocks. Nonsurgical and surgical options exist. These procedures recently have the highest growth rate among all cosmetic surgery procedures in the United States. 1 2 In this letter, we suggest a classification of gluteal augmentation procedures. The gluteal augmentation can broadly be classified into **pseudo-augmentation (illusionary)** and **real (true)** augmentation ( Fig. 1 ).

**Fig. 1:**
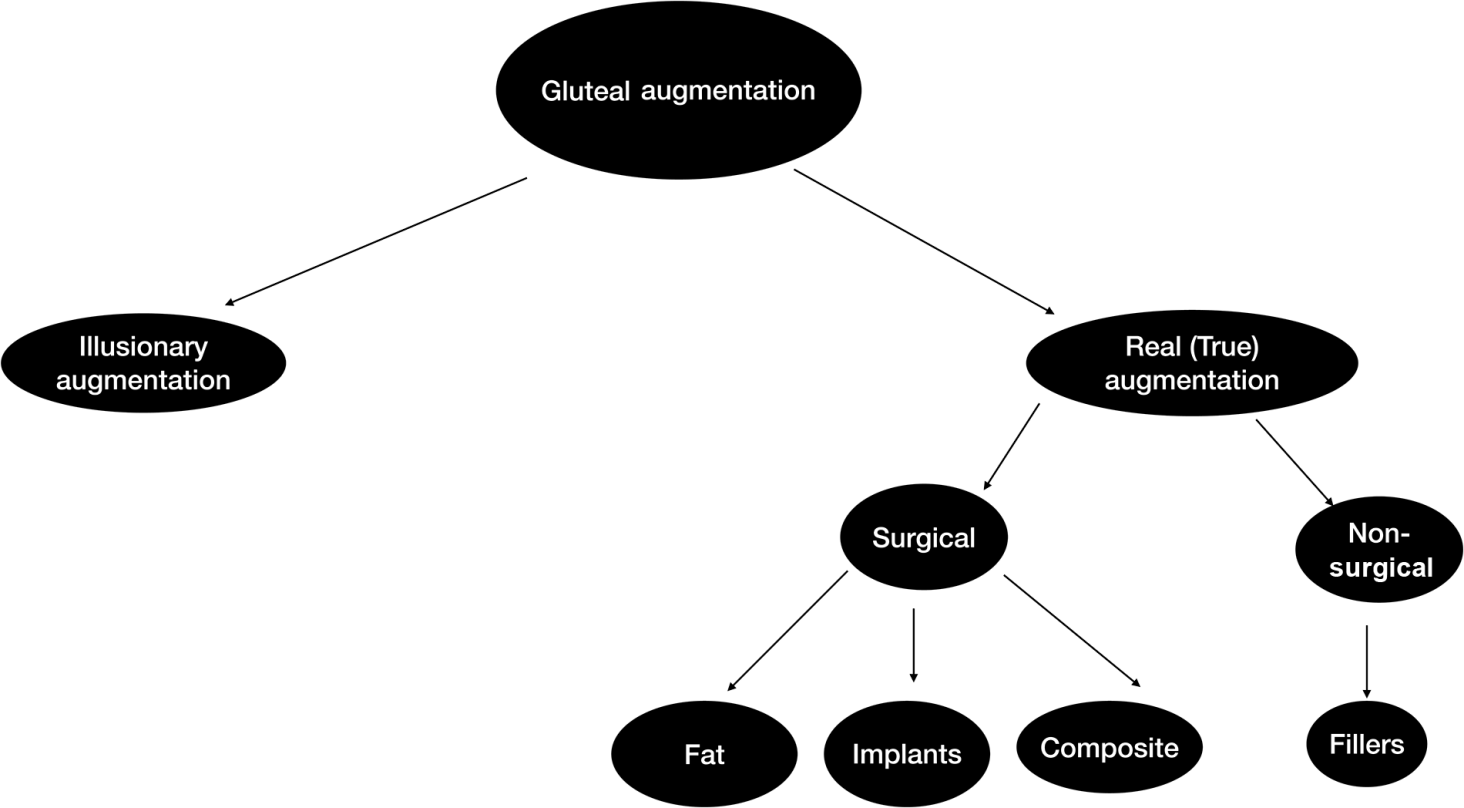
Diagram of the Sabri-Daoud gluteal augmentation classification.

- Pseudo-augmentation (or illusionary augmentation): It is the way by which the size of the buttocks appears to be altered, without actual alteration to the buttocks themselves. It consists of performing liposuction to the areas surrounding the gluteal region, such as the love handles and side saddles, to give the impression that the buttocks are bigger.
- Real (true) augmentation: It involves altering the actual size of the buttocks, via nonsurgical (aesthetic medicine act) or surgical means.
∘ Nonsurgical means of true gluteal augmentation is by the injection of various fillers into the gluteal region, such as Macrolane, HYAcorp, and others. The advantages of this method include that it is a minimally invasive procedure, with avoidance of surgery and general anesthetic risks. The disadvantages, however, include a significant risk of complications such as filler migration, inflammatory reaction, granuloma formation, and infection; that it is expensive; that the effects are often suboptimal and short-lived. 3
  ∘ Surgical methods of true gluteal augmentation include fat injection, gluteal implants, and composite gluteal augmentation.

1. *Fat injection* involves the process of liposuction and subsequent lipofilling to the gluteal region. The advantages of this method include the double-effect of fat removal in desired areas and volume gain in the buttocks region. The disadvantages include needing to have enough fat to perform the original liposuction; the high percentage of fat resorption within 6 months following the procedure which may apply a necessity to do more than one session to obtain the desired result, and the risk of fat embolism. 4 Sterodimas et al, 5 described stromal enriched lipograft, which increased and prolonged duration of the grafted fat that makes the necessity to repeat procedures very rare, as this technique allows to target the subcutaneous layer as the recipient site. 5
2. Gluteal implants are another method by which the buttocks may be truly augmented. They are long-lasting, can vary in size according to patient preference within the limit of implants size availability, and continue to hold their firmness over time. They have minimal associated disadvantages—pain that may radiate to lower limbs and it could continue for couple of weeks and needs to be managed by some analgesia and corticosteroid, the implant can be flipped over and that can be happened once or more than once, other complications like infection, seroma, wound healing complications, visible scars, and implant extrusion. 1 6
3. Composite gluteal augmentation involves the combination of gluteal implants and fat grafting. This technique is used to hide the implants, leading to a more natural and fuller look to the buttocks.

## Conclusion

To apply the treatment plan, we need to have a classification that makes the analysis of the case easier and applying the most adaptable procedure for each patient, according to patient's wish, facilities, technical skills of the practitioner, and availabilities of necessary materials and instruments.
